# Evaluation and Optimization of Protein Extraction From *E. coli* by Electroporation

**DOI:** 10.3389/fbioe.2020.543187

**Published:** 2020-09-08

**Authors:** Saša Haberl Meglič, Nika Janež, Matjaž Peterka, Karel Flisar, Tadej Kotnik, Damijan Miklavčič

**Affiliations:** ^1^Faculty of Electrical Engineering, University of Ljubljana, Ljubljana, Slovenia; ^2^Centre of Excellence for Biosensors, Instrumentation and Process Control, Centre for Biotechnology, Ajdovščina, Slovenia; ^3^Department of Biotechnology, Jožef Stefan Institute, Ljubljana, Slovenia

**Keywords:** protein extraction, *E. coli*, electroporation, glass–bead milling, ultrasonication, host DNA, endotoxin

## Abstract

Growing diversity of protein-based technologies dictates further development of bio manufacturing to lower the cost of production and maximize yields. Intracellularly expressed recombinant proteins must be extracted from production host prior to purification. Use of electroporation to obtain proteins from bacteria and yeasts has been demonstrated in several studies for different modes of operation and formats. Here we tested various protocols for protein extraction from *Escherichia coli* by means of electroporation. The tested protocols were compared to established extraction methods of ultrasonication and glass-bead milling in terms of protein yields and content of impurities such as host cell DNA and endotoxins in the lysate. Protein extraction yield was maximal when exponentially growing bacteria were treated at 37°C, regardless of the electroporation mode of operation (batch or flow). We were unable to eliminate co-extraction of host DNA and endotoxins, but with 8 × 1 ms, 5 kV/cm, 1 Hz pulses they were minimized. Yields with optimized electroporation (up to 86 g protein/kg dry weight) were inferior to those in ultrasonication (up to 144 g protein/kg dry weight) and glass-bead milling (up to 280 g protein/kg dry weight). Nevertheless, electroporation largely avoids cell lysis and disintegration with which the extract is a mix of extracted proteins with debris of the bacterial envelope and bacterial DNA, which necessitates further purification.

## Introduction

In the bio manufacturing of intracellular proteins, host cell envelope disruption is required to release the target recombinant proteins into the medium. The resulting lysate contains target proteins as well as impurities from host cells and growth medium which are then removed by purification. The type of extraction method affects the co-extraction of host cell components i.e., impurities, which is especially important in the production of protein therapeutics. Methods to open cell envelope and extract proteins are divided into mechanical and non-mechanical (Tan and Yiap, 2009). Mechanical methods like bead milling, high pressure homogenization, ultrasonication, cause cell lysis. Non-mechanical methods, chemical or enzymatic, are gentler, causing limited changes in cell envelope permeability resulting in outflow of intracellular content, but still have drawbacks, including expensive and often toxic chemicals, with their pharmaceutical production restricted by regulatory bodies.

*Escherichia coli* and other bacteria used as production hosts have the advantage of fast and easy cultivation as well as high recombinant protein production yields (Assenberg et al., 2013). Key microbial cell impurities affecting product’s safety and efficiency are endotoxins (lipooligosaccharides), host cell DNA and host cell proteins. Endotoxins are a potent pyrogenic compound causing strong immunogenic response in mammals and must be reduced to concentration below 5 EU/kg/hr according to European Pharmacopoeia^1^. Host cell DNA contributes to increased viscosity of lysate resulting from cell disruption, impedes purification processes, and carries potential risk for human health (Stone et al., 2018). Host cell proteins, produced by the host to sustain normal cell functions, may be immunogenic, toxic or active in human bodies, and if they possess proteolytic activity, they may cause degradation of the recombinant protein (Goey et al., 2018).

Protein extraction by means of electroporation has been implemented to date in batch and flow modes of operation, as well as on microchips to assist protein research (Shiina et al., 2004; Matos et al., 2013; Flisar et al., 2014; Haberl Meglic et al., 2015). The extraction mechanism is based on induction of changes in cell envelope permeability due to exposure to sufficiently strong electric pulses (Kotnik et al., 2010). The choice of electric pulse parameters depends on the desired effect on bacteria, where a suitable choice of amplitude, duration and number of electric pulses delivered is of key importance (Kotnik et al., 2015). Longer electric pulses with lower amplitudes have been demonstrated to give rise to higher protein yields without severely compromising cell viability, and are therefore considered optimal for protein extraction (Haberl Meglic et al., 2015). Even though electroporation can also result in loss of cell viability, the damage is in general more limited than in chemical cell lysis, which is advantageous for extraction, as impurities are not as extensively co-extracted. For Gram-negative bacteria, electroporation efficiency was found to depend on the inherent properties of bacterial cell envelope and differs on a species and strain level (Wirth et al., 1989). Inner membrane integrity depends on culture medium, cultivation mode, growth rate and growth phase (Chou, 2007). These host envelope properties were also found to affect cell’s susceptibility to electric pulses and protein extraction efficiency (Coustets et al., 2015; Haberl Meglic et al., 2015). Protein extraction by means of electroporation was found to increase enzyme activity and production yields in fed-batch processes compared to traditional extraction methods (Shiina et al., 2004; Coustets et al., 2015).

Here, we optimized extraction temperature and bacterial growth phase to maximize protein yield and survival of bacteria treated with electroporation. We further evaluated and compared extractions by electroporation with two established methods ultrasonication and glass-bead milling in terms of protein yield and co-extraction of impurities. Additionally, we compared electroporation in batch and continuous flow mode. This mode of operation has gained importance in protein manufacturing for the production of new products, labile biologics and those with uncertain demand (Hernandez, 2017).

## Materials and Methods

### Bacterial Strains and Cultivation

Two ***E*. *coli*** TOP10 (K12 derivative) variants were used in this study: the kanamycin-resistant TOP10 pEGFP-N1 and the ampicillin-resistant TOP10 pUC19-hMGFP (both from Clontech Laboratories Inc., Mountain View, CA, United States). Strains were grown in LB medium (Sigma-Aldrich Chemie GmbH, Schnelldorf, Germany) supplemented by 50 μg/ml kanamycin (Carl ROTH Gmbh, Germany) for TOP10 pEGFP-N1 or 100 μg/ml ampicillin (Sigma-Aldrich Chemie GmbH) for TOP10 pUC19-hMGFP. Bacteria for extraction were prepared by shake flask cultivation at 37°C, 200 rpm. Based on the pre-determined growth curve (Haberl-Meglič et al., 2016), bacteria were harvested at appropriate growth phase by centrifugation (4,248 × **g**, 30 min, 4°C) and resuspended to OD600 2.1 corresponding to about 10^9^ colony forming units per milliliter (CFU/ml).

**FIGURE 1:**
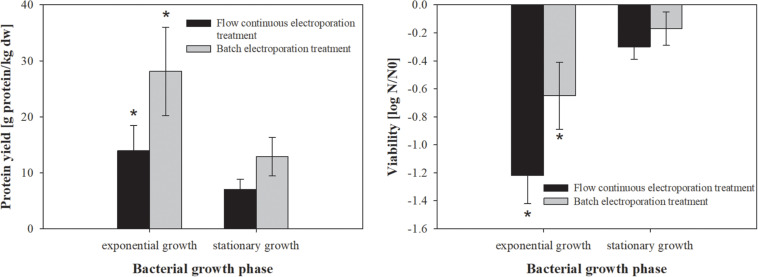
The effect of bacterial growth phase on protein extraction **(left)** and bacterial viability **(right)**. Bacterial cells (*E. coli* TOP10 pEGFP-N1) were grown to exponential (5 h growth time; treated mass of cells was 2.968 × 10^–7^ kg dw) and stationary (10 h growth time; treated mass of cells was 9.38 × 10^–7^ kg dw) growth phase, incubated for 30 min at 4°C and exposed to two different electroporation treatments: (black column) flow continuous electroporation treatment where each bacterial cell was subjected to eight pulses with 5 kV/cm of electric field strength, 1 ms pulse duration and 10 Hz of repetition frequency; (gray column) batch electroporation treatment where eight pulses were applied with 5 kV/cm of electric field strength, 1 ms pulse duration and 1 Hz of repetition frequency. After the treatment bacterial cells were incubated for 1 h at 4°C. Values represent means (all tests were performed in triplicate) and error bars are determined from standard deviation. Asterisk (*) represents statistically significant (*p* < 0.05) difference versus stationary growth phase.

**FIGURE 2:**
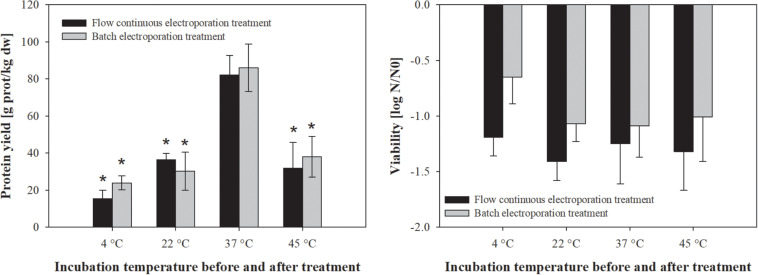
The effect of incubation temperature before and after treatment on protein extraction **(left)** and bacterial viability **(right)**. Bacterial cells (*E. coli* TOP10 pEGFP-N1) in exponential growth phase were incubated before (30 min) and after treatment (1 h) at different temperatures (4, 22, 37, and 45°C) and exposed to two different electroporation treatments: (black column) flow continuous electroporation treatment where each bacterial cell was subjected to eight pulses with 5 kV/cm of electric field strength, 1 ms pulse duration and 10 Hz of repetition frequency; (gray column) batch electroporation treatment where eight pulses were applied with 5 kV/cm of electric field strength, 1 ms pulse duration and 1 Hz of repetition frequency. Treated mass of cells was 2.532 × 10^–7^ kg dw. Values represent means (all tests were performed in triplicate) and error bars are determined from standard deviation. Asterisk (*) represents statistically significant (*p* < 0.05) difference versus incubation temperature at 37°C.

**FIGURE 3:**
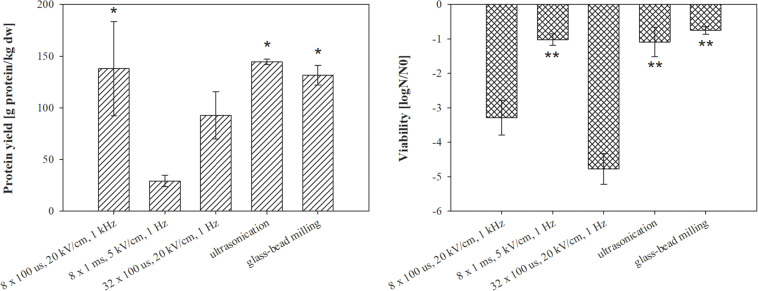
The effect of different treatments on protein extraction **(left)** and bacterial viability **(right)**. Bacterial cells (*E. coli* TOP10 pEGFP-N1) in exponential growth phase were incubated before and after treatment at 37°C and exposed to three different treatments: batch electroporation treatments, ultrasonication and glass-bead milling. Treated mass of cells was 2.422 × 10^–7^ kg dw. Values represent means (all tests were performed in triplicate) and error bars are determined from standard deviation. Asterisk (*) represents statistically significant (*p* < 0.05) difference versus electroporation protocols 8 × 1 ms, 5 kV/cm, 1 Hz and 32 × 100 μs, 20 kV/cm, 1 Hz. Two asterisk (**) represents statistically significant (*p* < 0.05) difference versus electroporation protocols 8 × 100 μs, 20 kV/cm 1 kHz and 32 × 100 μs, 20 kV/cm, 1 Hz.

**FIGURE 4:**
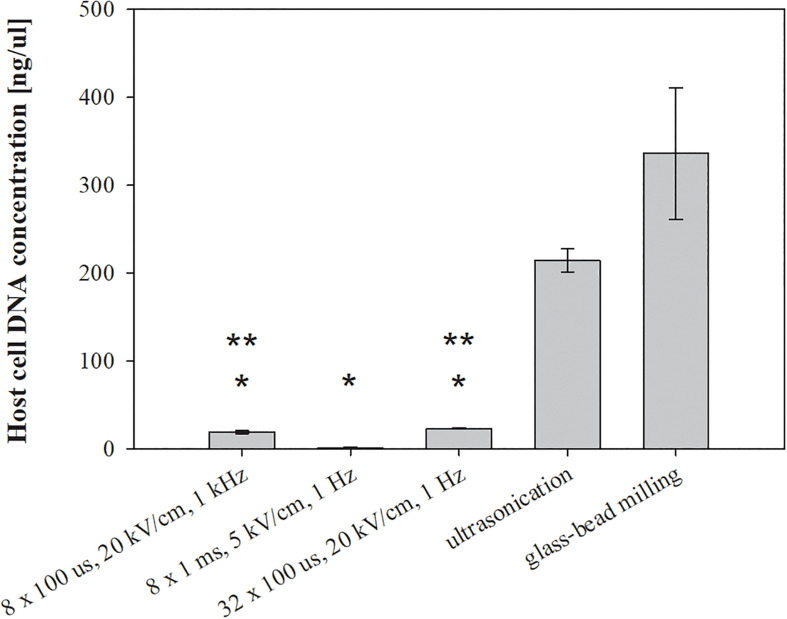
The effect of different treatments on host DNA leakage. Bacterial cells (*E. coli* TOP10 pEGFP-N1) in exponential growth phase were incubated before and after treatment at 37°C and exposed to three different treatments: batch electroporation treatments, ultrasonication and glass-bead milling. Treated mass of cells was 2.072 × 10^–7^ kg dw. Values represent means (all tests were performed in triplicate) and error bars are determined from standard deviation. Asterisk (*) represents statistically significant (*p* < 0.05) difference versus ultrasonication or glass-bead milling. Two asterisk (**) represent statistically significant (*p* < 0.05) difference versus electroporation protocol 8 × 1 ms, 5 kV/cm, 1 Hz.

**FIGURE 5:**
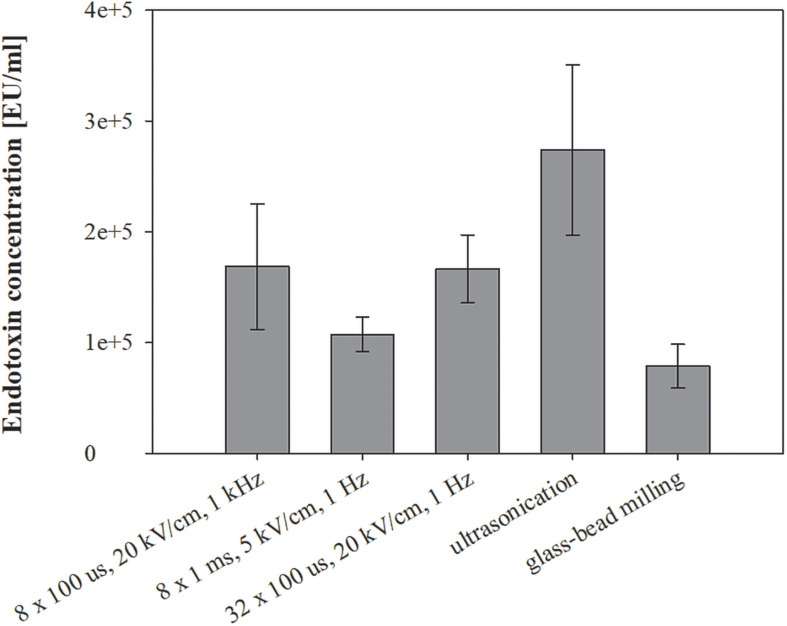
The effect of different treatments on endotoxin leakage. Bacterial cells (*E. coli* TOP10 pEGFP-N1) in exponential growth phase were incubated before and after treatment at 37°C and exposed to three different treatments: batch electroporation treatments, ultrasonication and glass-bead milling. Endotoxin Units (EU) is a measure of the activity of endotoxin and one EU is approximately equivalent to 100 pg of *E. coli* lipopolysaccharide – the amount present in approximately 105 bacteria. Treated mass of cells was 2.498 × 10^–7^ kg dw. Values represent means (all tests were performed in triplicate) and error bars are determined from standard deviation.

**FIGURE 6:**
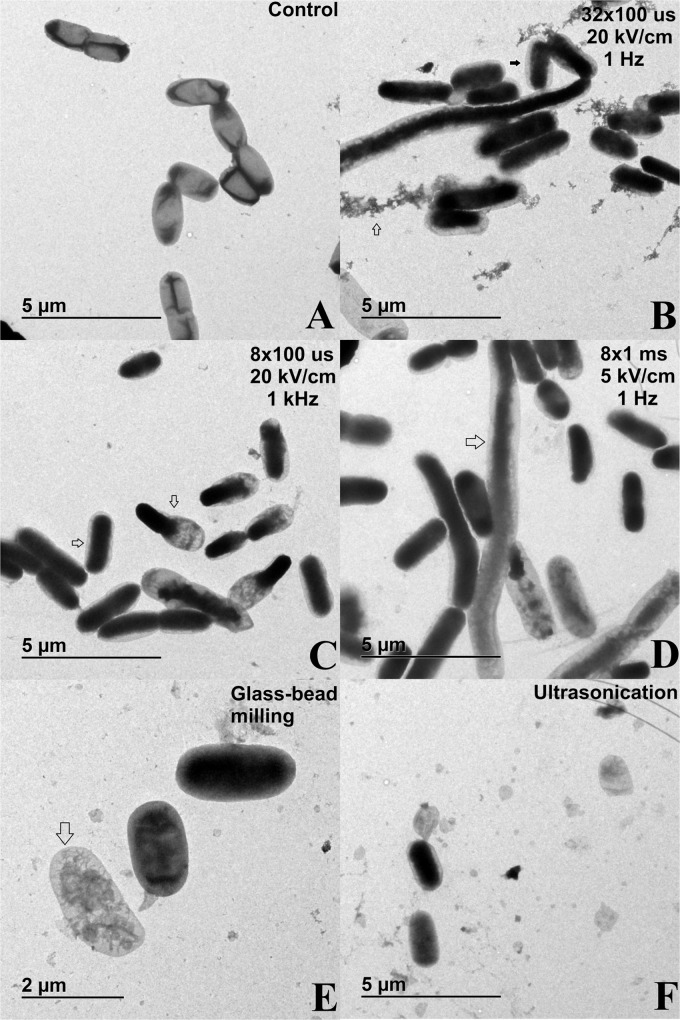
TEM images of bacterial cells (*E. coli* TOP10 pEGFP-N1) subjected to different treatment: **(A)** control sample – bacterial suspensions of *E. coli* TOP10 pEGFP-N1 that were not subjected to extraction methods but were otherwise treated in the same way as experimental samples; **(B)** electric pulses 32 × 100 μs, 20 kV/cm, 1 Hz; **(C)** electric pulses 8 × 100 μs, 20 kV/cm, 1 kHz; **(D)** electric pulses 8 × 1 ms, 5 kV/cm, 1 Hz; **(E)** glass–bead milling; **(F)** ultrasonication. Bacterial cells in exponential growth phase were incubated before and after treatment at 37°C.

*Escherichia coli* TOP10 pEGFP-N1 strain was used for: (i) optimizing electroporation protocol (to assess the effect of bacterial growth phase and pre-/post-pulse incubation temperature) (see Figures 1, 2); (ii) assessing scalability of electroporation (continuous flow electroporation extraction) (see Figures 1, 2); (iii) assessing the effect of different treatments on total protein extraction and co-extraction of unwanted compounds (endotoxins, host DNA) (see Figures 3–5); and (iv) assessing bacterial morphology after different treatments (see Figure 6).

**FIGURE 7:**
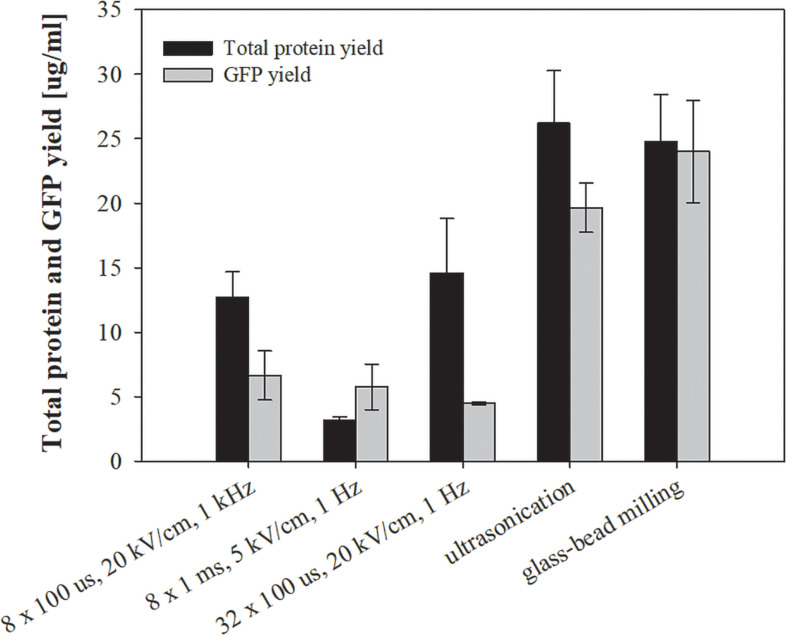
The effect of different treatments on total protein and specific (GFP) protein extraction. Bacterial cells *E. coli* TOP10 pUC19-hMGFP in exponential growth phase were incubated before and after treatment at 37°C and exposed to three different treatments: batch electroporation treatments, ultrasonication and glass–bead milling. Values represent means (all tests were performed in triplicate) and error bars are determined from standard deviation.

*Escherichia coli* TOP10 pUC19-hMGFP strain carries GFP protein which was chosen as model target protein, because its fluorophore gives stable signal in various media due to its tight and stable structure. Therefore, this strain was used only to show the difference in extraction of model target protein by different methods (electroporation, ultrasonication, and glass-bead milling) (see Figure 7).

### Assessing the Effect of Bacterial Growth Phase and Pre-/Post-Pulse Incubation Temperature on Protein Extraction by Means of Batch or Flow Continuous Electroporation Treatment

Bacterial culture TOP10 pEGFP-N1 was in first set of experiments grown to early exponential phase (5 h) or stationary growth phase (10 h), harvested as described above (see section “Bacterial Strains and Cultivation”) and chilled to 4°C for 30 min before the electric pulse application. After the treatment, bacterial suspension was again chilled to 4°C for 1 h.

In second set of experiments bacterial culture TOP10 pEGFP-N1 was grown to early exponential phase (5 h), harvested as described above (see section “Bacterial Strains and Cultivation”) and aliquoted for following parallel experiments. Each aliquot was incubated at a different temperature (4, 22, 37, 45°C) for 30 min prior to electroporation.

After the incubation, bacteria were exposed to batch or flow continuous electroporation treatment and then again incubated at a different temperature (4, 22, 37, 45°C) for 1 h.

In batch electroporation treatment, 140 μl of bacterial suspension was placed between stainless steel plate electrodes (1 mm gap) and electroporated by a square wave electric pulse generator HVP – VG (IGEA s.r.l., Italy) using 8 rectangular pulses with duration of 1 ms, electric field strength 5 kV/cm, and repetition frequency 1 Hz. These electroporation parameters were used as they were previously found efficient for protein extraction (Haberl Meglic et al., 2015).

In flow continuous electroporation treatment, laboratory scale flow system with square wave prototype pulse generator and treatment chamber (Supplementary Figure 1) was used. Flow treatment chamber has a cross section area of 2.5 × 2.0 mm and is 10 cm long, with an inter electrode gap (stainless steel electrodes) of 2.5 mm (Supplementary Figure 1). Bacteria were subjected to a train of 8 rectangular pulses with duration of 1 ms, electric field strength 5 kV/cm (500 V) and repetition frequency 10 Hz. The flow velocity through the chamber was adjusted as to expose each bacterial cell to eight electrical pulses as in batch extraction experiments – 50 ml of bacterial suspension was loaded into the flow treatment chamber at 37.5 ml/min using a peristaltic pump. The lysate was analyzed after a single passage through the continuous flow cell. Prior to the treatment, the tubing and the continuous flow treatment chamber were flushed with 70 % ethanol and sterile distilled water. Extraction by flow system was compared to batch extraction to evaluate effect of the growth phase and of the pre- and post-pulse temperature on extraction outcome.

Treatment was repeated at least twice on separate occasions using fresh bacterial suspensions until adequate lysate volume was obtained. Electric field strength was estimated as *E = U/d*, where *U* denotes applied voltage and *d* inter-electrode distance.

As control, we used bacterial suspensions that were not subjected to electroporation but were otherwise treated in the same way as experimental samples.

### Assessing the Effect of Electroporation Parameters, Ultrasonication and Glass-Bead Milling

Bacterial culture TOP10 pEGFP-N1 was grown to early exponential phase (5 h), harvested as described in see section “Bacterial Strains and Cultivation” and incubated for 30 min at 37°C prior the extraction.

Extraction by means of electroporation was carried out in batch system, using following electroporation parameters: (i) a train of 8 rectangular pulses of 100 μs, electric field strength 20 kV/cm (2000 V) and repetition frequency 1 kHz, (ii) a train of 8 rectangular pulses of 1 ms, 5 kV/cm (500 V) 1 Hz, and (iii) a train of 32 rectangular pulses of 100 μs, 20 kV/cm (2000 V), 1 Hz.

The energy input was calculated using the formula below, where *U* is applied voltage, *I* electric current, *n* number of applied pulses, *T* pulse duration and *V* sample volume. The results are presented in Table 1.

$$W=\frac{U\cdot I\cdot n\cdot T}{V}$$

**TABLE 1:** Calculated energy input delivered by each set of pulse parameters in batch extraction of proteins from *E. coli*.

Operation mode	Electroporation pulse parameters	Energy input (kJ/L)
Batch	8 × 100 μs, 20 kV/cm, 1 kHz	65
	8 × 1 ms, 5 kV/cm, 1 Hz	37
	32 × 100 μs, 20 kV/cm, 1 Hz	379
Continuous	8 × 1 ms, 5 kV/cm, 10 Hz	37

Extraction by means of ultrasonication was performed with Ultrasonic homogenizer 4710 (Cole Parmer Instrument Co, Vernon Hills, IL, United States). 3 ml of bacterial suspension was sonicated three times for 20 s at 90 W amplitude and 25 Hz frequency. Samples were kept on ice while sonicated to prevent heating.

Extraction by means of glass-bead milling was carried out by mixing 1.2 ml of bacterial suspension and 0.1 mm glass-beads at approximate ratio 1:1. Homogenization was performed for 5 min at 2850 rpm using bead beater Digital Disruptor Genie (Scientific Industries Inc., Bohemia, NY, United States).

The bacterial suspension was after all treatments incubated for 1 h at 37°C. As control, we used bacterial suspensions that were not subjected to extraction methods but were otherwise treated in the same way as experimental samples.

### Assessing Total and Target (GFP) Proteins

Total protein extraction was determined with assay based on the Bradford Reagent (Sigma-Aldrich Chemie GmbH, Germany) (BSA) and was performed according to the manufacturer’s recommendations. This reagent is suitable for 1–10 μg/ml micro assays. Calibration curve was prepared from serial dilutions of bovine serum albumin of known concentration. Samples for total protein quantification were sterile filtered through a 0.22 μm filter (Millex-GV; Millipore Corporation, Billerica, MA, United States) immediately after the post-treatment incubation. Final concentration of extracted proteins in the sample was determined after the protein concentration in the control was subtracted from the initial protein concentration in the sample. Protein concentration was expressed as grams of protein per kilogram of dry weight (abbreviated in the Results as g protein/kg dw).

Samples for GFP quantification were clarified after the post-treatment incubation by centrifugation 7155 × *g*, 5 min, 4°C. The supernatants were collected, and pellets were gently re-suspended in the sterile distilled water. Fluorescence was measured in the pellets and supernatants immediately after extraction. Detection of GFP was carried out on spectrofluorometer (Tecan infinite M200, Tecan, Austria) at excitation wavelength 490 nm and emission wavelength 520 nm. Concentration of GFP was estimated from the calibration curve, and as a standard, purified GFP of known concentration was used. Limit of detection was estimated at 17 μg GFP/ml and limit of quantification 22 μg GFP/ml.

### Bacterial Viability Assessment

Immediately after the post-treatment incubation, serial dilution of 50 μl sample aliquots in 0.9% NaCl was made. One hundred microliters of each dilution were plated onto the LB agar supplemented by suitable antibiotic (see section “Bacterial Strains and Cultivation”) and incubated for 24 h at 37°C. From the bacterial counts’ CFU/ml were calculated. The bacterial cell number reduction was expressed as log (*N*/*N*_0_), where *N* represents the number of CFU/ml in treated sample and *N*_0_ the number of CFU/ml in the control sample.

### Assessing Host Cell DNA Extraction After Different Treatments

Host cell DNA was quantified by real time quantitative PCR (qPCR) analysis based on amplification of 16 rRNA gene developed by external service provider NIB (Ljubljana, Slovenia). As reference material, purified and restricted *E. coli* DH5α DSM 6897 genomic DNA was used, since both bacteria belong to the same species and have the same number of rRNA copies in their genome (Durfee et al., 2008; Anton and Raleigh, 2016). Samples for qPCR analysis were sterile filtered through a 0.22 μm filter immediately after the post-treatment incubation and kept at –20°C until analysis.

### Assessing Endotoxin Extraction After Different Treatments

Endotoxin content was measured using chromogenic limulus amoebocyte assay (LAL assay) by external service provider Jafral d.o.o. (Ljubljana, Slovenia). Samples for LAL assay were sterile filtered through a 0.22 μm filter immediately after the post-treatment incubation and kept at 4°C until analysis.

### Assessing Bacterial Morphology After Different Treatments

Morphology of bacteria after different treatment methods was assessed by transmission electron microscopy – TEM (Philips CM 100, Philips Electronics, Amsterdam, Netherlands). The cells were applied to Formvar/carboned 400-mesh copper grids immediately after the treatment without post incubation and were negatively stained by 1% uranyl acetate.

### Statistical Analysis

Experiments were repeated three or more times, on different days to prove repeatability. Results were evaluated using an unpaired *t*-test analysis (SigmaPlot 11.0, Systat Software, Richmond, CA, United States) and were considered as statistically different at *p* < 0.05.

## Results

### Effect of Bacterial Growth Phase, Pre- and Post-Treatment Temperature on Protein Extraction by Means of Batch or Flow Continuous Electroporation Treatment

Bacterial growth phase strongly affects bacterial metabolism as well as the ratio of protein to lipid content in the membrane, which also affects the efficiency of electroporation (Haberl-Meglič et al., 2016). Therefore, we firstly assessed the effect of bacterial growth phase on protein extraction by means of electroporation in batch and flow continuous system (Figure 1). The highest protein yield was obtained from bacteria in exponential growth phase (14 ± 4.4 g prot/kg dw), compared to stationary phase (7 ± 1.8 g prot/kg dw), but the stationary growth phase exhibited lower viability loss (Figure 1). There was no statistically significant difference in protein extraction or viability loss between flow continuous and batch electroporation treatment for either bacterial growth phase.

Based on our results bacterial cells were most susceptible to electric pulses (more proteins were extracted) in earlier stages of growth (exponential growth phase).

Temperature has a significant effect on cell membrane structure and by that on permeabilization of the cell membrane. Thus, we secondly assessed the effect of different incubation temperatures on protein extraction by means of electroporation in batch and flow continuous system (Figure 2). The increase in pre- and post-pulse temperature resulted in improved protein yield, with the highest protein concentration obtained at 37°C (82 ± 10.6 g prot/kg dw), followed by 22°C (36.4 ± 3.4 g prot/kg dw), 45°C (31.8 ± 13.9 g prot/kg dw) and 4°C (15.3 ± 4.6 g prot/kg dw) for batch and flow continuous electroporation treatment (Figure 2). There was no statistically significant effect of different incubation temperatures on viability when bacterial cells were electroporated (Figure 2).

Our results show that the higher amount of extracted proteins can be achieved when bacterial cells are electroporated in exponential growth phase (Figure 1) and incubated at 37°C (Figure 2). Therefore all the following experiments were performed at these conditions.

The efficiency of extraction by means of electroporation did not differ when performed in batch or flow continuous treatment mode, thus scaling up for protein extraction seems achievable.

### Comparison of Extraction of Proteins by Means of Electroporation, Ultrasonication and Glass-Bead Milling

Our motivation in this part of the study was to compare extraction by means of electroporation with two most widely used methods for protein extraction. Moreover, different electroporation parameters (electric field strength, pulse duration, pulse amplitude, and repetition frequency) strongly affect the magnitude of membrane permeabilization and by that also extraction yield. Therefore, three different electroporation protocols were tested. The lysates obtained from *E. coli* TOP10 pEGFP-N1 by the different extraction methods were compared in terms of total protein content, bacterial viability after the treatment (Figure 3), and co-extracted undesired molecules in lysate (host cell DNA-Figure 4 and endotoxins-Figure 5). Here, total protein content was used to describe overall protein yield and to observe differences between proteins extracted by tested extraction methods, although this parameter commonly reflects proteinaceous impurities in the protein therapeutics. Bacteria *E. coli* TOP10 pEGFP-N1 were harvested at early exponential phase (5 h) and incubated for 30 min at 37°C prior treatment.

The highest total protein yield was obtained by ultrasonication (144.4 ± 2.6 g prot/kg dw) followed by electroporation protocol 8 × 100 μs, 20 kV/cm, 1 kHz (137.9 ± 45.6 g prot/kg dw), glass-bead milling (131.6 ± 9.5 g prot/kg dw), and electroporation protocols 32 × 100 μs, 20 kV/cm, 1 Hz (92.6 ± 22.9 g prot/kg dw), 8 × 1 ms, 5 kV/cm, 1 Hz (29.2 ± 5.4 g prot/kg dw) (Figure 3, left). Considering the protein concentration in the electroporated lysates, we obtained 50 ± 12% proteins by 100 μs electric pulses and 12 ± 6% with pulse parameter 8 × 1 ms, 5 kV/cm, 1 Hz. Protein yields and viability of extraction replicates varied, but the measurement trends were clear.

As it can be seen in Figure 3-left, electroporation can be effectively used for protein extraction; moreover, different electroporation parameters affect the protein yield. As expected, electroporation protocol 32 × 100 μs, 20 kV/cm, 1 Hz most strongly affected bacterial viability (Figure 3-right) due to higher energy input (379 kJ/L) (Novickij et al., 2018). Nevertheless protein yields were lower compared to electroporation protocol where lower energy of the pulses was applied – 65 kJ/L (8 × 100 μs, 20 kV/cm, 1 kHz), which suggests that the risen temperature during the treatment possibly damaged proteins. With electroporation protocol 8 × 100 μs, 20 kV/cm, 1 kHz, a similar amount of proteins as with two standard protein extraction methods (ultrasonication, glass-bead milling) was obtained.

The viability loss of treated bacteria was lower after glass-bead milling, electroporation protocol of 8 × 1 ms, 5 kV/cm, 1 Hz, and ultrasonication (Figure 3-right). Since we predicted that bacterial viability was associated with the release of unwanted molecules, we checked the amount of extracted host DNA and endotoxins. The bacterial viability loss was, however, not congruent with the measured concentration of host cell DNA. The highest concentration of host cell DNA was detected in the lysates obtained by glass-bead milling (336.34 ± 74.73 ng/μl) and ultrasonication (214.34 ± 13.35 ng/μl), more than ten times higher as in lysates obtained by means of electroporation protocols 32 × 100 μs, 20 kV/cm, 1 Hz (23.36 ± 0.66 ng/μl) and 8 × 100 μs, 20 kV/cm, 1 kHz (19.33 ± 1.65 ng/μl) (Figure 4).

The lowest concentration of co-extracted host cell DNA, below 2 ng/μl (1.28 ± 0.73 ng/μl), was obtained with pulse parameters 8 × 1 ms, 5 kV/cm, 1 Hz (Figure 4). Our results implies that selection of appropriate electroporation protocol is important in order to avoid also co-extraction of unwanted host DNA.

Endotoxins are considered contaminants in lysate and were co-extracted at elevated levels by all tested extraction methods (Figure 5). The highest amount of endotoxins was extracted with ultrasonication (274167 ± 76977 EU/ml), followed by electroporation protocols 8 × 100 μs, 20 kV/cm, 1 kHz (168537 ± 56659 EU/ml), 32 × 100 μs, 20 kV/cm, 1 Hz (166485 ± 30772 EU/ml), 8 × 1 ms, 5 kV/cm, 1 Hz (107401 ± 15669 EU/ml) and glass-bead milling (78808 ± 19775 EU/ml). Biological replicate measurements fluctuated and masked the trends in the endotoxin level of co-extraction in this study. The post-pulse incubation at 37°C did not affect the co-extraction of endotoxins, and neither did the growth in the media with or without antibiotic (data not shown).

TEM was used to assess the effect of the methods on bacterial morphology. Irrespective of extraction method, we observed that treated bacteria have an enlarged periplasmic space, which indicates that outer membrane was interrupted (Figure 6). Cell debris was evident after electroporation protocol of 32 × 100 μs, 20 kV/cm, 1 Hz, as well as after glass–bead milling and ultrasonication (Figures 6B,E,F), which implies that these methods for protein extraction are the most destructive for the bacteria. Additionally, bacterial ghost-like structures and elongated bacterium-like structures were present in all analyzed lysates. The effect of electric pulses on bacterial morphology is similar among tested parameters, but the extent of damage differs, with the protocol of 8 × 1 ms, 5 kV/cm, 1 Hz causing the mildest effect (Figure 6D).

### Extraction of GFP by Electric Pulses, Glass-Bead Milling and Ultrasonication

The above tested extraction methods were further utilized to extract specific protein (GFP) from *E. coli* TOP10 pUC19-hMGFP. We have chosen this bacteria, since it is constitutively expressing GFP. The viability and level of impurities for this bacteria did not differ from the above presented measurements, thus validating them (data not shown). The highest concentration of GFP was measured in the lysates obtained by glass-bead milling (24 ± 3.96 μg/ml), and the second highest by ultrasonication (19.65 ± 1.91 μg/ml) (Figure 7). Although the amount of extracted GFP was with electroporation protocols lower compared to glass-bead milling or ultrasonication, selectivity for GFP extraction was observed with the protocol of 8 × 1 ms, 5 kV/cm, 1 Hz, where GFP concentration remained as high as with other two parameters, while total protein content was notably lower (see Figure 7).

## Discussion

Protein extraction from *E. coli* by electroporation is based on transient permeabilization of cell envelope, allowing the outflow of cytoplasmic content to the medium without causing cell disintegration. Based on our previous work (Haberl Meglic et al., 2015) we focused here on optimization of the extraction protocol and evaluation of protein extraction by electroporation in comparison to glass-bead milling and ultrasonication as two established methods that generally lead to total cell disintegration. Superior protein yield was obtained from bacteria in exponential growth phase regardless of the electroporation mode of operation (batch, flow) (Figure 1). Inner membrane of Gram-negative bacteria in exponential growth phase is known to be more permeable than in stationary phase, probably causing increased susceptibility to heat, antimicrobial agents and electric pulses (Coustets et al., 2015; Pletnev et al., 2015; Figure 1). It was also previously shown that exponential and stationary bacteria also significantly differ in their resting membrane potential values (Bot and Prodan, 2010). Membrane fluidity (viscosity) is also almost instantly affected by large fluctuations in environmental temperature, since cooling causes lipids to pack closely together and increases membrane rigidity, while heating causes the opposite effects (Mika et al., 2016). This apparently causes the electropermeabilized membrane to reseal slower at lower temperatures, thus prolonging the leakage of the intracellular content into the medium (Xie and Tsong, 1992). However, we observed that higher pre- and post-treatment temperatures are favorable for extraction of proteins regardless of the electroporation mode of operation (batch, flow), possibly due to higher membrane viscosity facilitating the permeabilization or hindering the resealing (Figure 2). Protein extraction yield and viability fluctuations among the extraction replicates can be attributed to large heterogeneity among bacteria in terms of membrane viscosity and other physical parameters of the bacteria observed elsewhere (Mika et al., 2016). The efficiency of extraction by means of electroporation did not differ significantly when performed in batch or continuous mode of operation, thus demonstrating its flexibility. As electroporation has been successfully used in an industrial pilot unit to pasteurize liquid foodstuff, scaling up in protein production seems feasible and viable (Picart-Palmade et al., 2019).

Using electroporation pulses differing in length, repetition frequency and amplitude, we achieved substantial differences in overall protein yield rather than a selective extraction of group of proteins with similar physical-chemical properties (Figures 3, 7). Absolute values of protein concentration in this study may be underestimated, because the lysates contain a wide variety of proteins with different dye responses, in contrast to the protein standard (BSA) – used for determining protein concentration (see section “Assessing Total and Target (GFP) Proteins”) – that has stable and unusually large dye response. Thus, lower signals may be falsely interpreted as lower concentrations. We observed also that GFP yield, estimated from fluorescence measurements, dropped when bacterial culture was treated by pulse parameter where higher energy was applied – 32 × 100 μs, 20 kV/cm, 1 Hz (Figure 7). Loss of fluorescence in tight structure of GFP is usually associated with denaturation that could be due to Joule heating.

Cell debris indicating cell envelope disintegration was notable after the glass–bead milling and ultrasonication as expected (Figures 6E,F), while after electroporation the highest amount of host cell DNA was co-extracted with 32 × 100 μs, 20 kV/cm, 1 Hz (Figure 4). The host cell DNA level in the lysates obtained by ultrasonication and glass-bead milling was likely overestimated, as viability loss is caused by lysis, and consequently host DNA is released. But viability was incongruent with released DNA, probably due to DNA fragmentation upon release to the medium and its accessibility to DNA polymerase in qPCR assay, resulting in concentration overestimation. Irrespective of the extraction method, the treated bacteria had an enlarged periplasmic space, which could also be a consequence of post-osmotic stress (Figure 6). Additionally, bacteria were resuspended in sterile distilled water, which enhances the stress and hinders cell recovery after the treatment. Modifications of electroporation medium could be important in bacterial survival, as it was shown before that by changing medium pH or supplementing it with reagents, both the electroporation efficiency and the host cell survival were improved (Garcia et al., 2007; Coustets et al., 2015). Beside changes in inner membrane permeability discussed above, outer membrane was often interrupted at one of the poles, thus enabling physical separation of cell wall and inner membrane, resulting in ghost like structures (Figure 6). The importance of these structures remains unknown in the context of extraction, but they have applications in other biotechnical fields (Langemann et al., 2010). The elongated bacterium-like structures were also observed, and these cannot be attributed to sample preparation, neither could they arise from growth, as bacteria were analyzed immediately after the extraction and prior to incubation. These structures suggest that bacterial fusion is happening during exposure to external electric field, as already observed but never examined in detail (Tyurin et al., 1997). We hypothesize that the observed cell wall ruptures at the poles may facilitate bacterial fusion by exposing the parts of the cell envelope that were reported to enable fusion of bacterial protoplasts (Gokhale et al., 1993). Bacterial fusion was historically used as a method to transfer genetic material between bacteria, and fusion by electric pulses has been suggested as a mechanism of horizontal gene transfer during early evolution when other mechanisms for such transfer did not yet exist or were still evolving (Kotnik, 2013).

Endotoxins were co-extracted in elevated concentrations regardless of the extraction method and independently from the cultivation with antibiotics (Figure 5). However, the lowest amount of endotoxins was obtained with glass-bead milling. Growing bacteria are known to constantly release endotoxins into the environment both *in vivo* and *in vitro*. This shedding is enhanced when bacterial culture is exposed to antibiotics, but the cells survive even when deprived of 40% of the lipopolysaccharide (LPS) layer – also known as lipoglycan layer or endotoxin layer (Marvin et al., 1989; Crosby et al., 1994). Since extraction mechanisms differ among tested methods, the endotoxins may be released into the medium in various physical-chemical forms, thus differently exposing the biologically active moiety. Namely, endotoxin is a large molecule consisting of a lipid A and polysaccharides (inner and outer core, and O-antigen). It has been shown that free endotoxins shed into the medium enhance the exposure of lipid A, which consequently results in stronger activation of LAL assay (assay for determining endotoxin levels in our study) components than purified endotoxin used as standard (Mattsby-Baltzer et al., 1991). This leads to interpretation of stronger signals as higher endotoxin concentrations, and absolute endotoxin content can thus be determined only by additional chemical analysis.

Total protein content, co-extraction of host cell DNA and endotoxins, and viability loss during electroporation of *E. coli* K12 carrying two different types of plasmids (pEGFP-N1 cloning vector vs. pUC19-hMGFP expression plasmid) were consistent (data not shown). Although recombinant protein yields (GFP) obtained with electroporation are not superior to ultrasonication or glass-bead milling, to certain degree, selectivity for GFP extraction was observed with the protocol of 8 × 1 ms, 5 kV/cm, 1 Hz, where GFP concentration remained as high as with other two electroporation parameters, while total protein content was notably lower (Figure 7). Energy consumption using the electroporation protocol of 32 × 100 μs, 20 kV/cm, 1 Hz was ten times larger than for 8 × 100 μs, 20 kV/cm, 1 kHz (Table 1). In terms of energy input, the most efficient extraction of total proteins was obtained by 8 × 100 μs, 20 kV/cm, 1 kHz and the highest GFP yield was for 8 × 1 ms, 5 kV/cm, 1 Hz.

In general, though total protein yields obtained by electroporation are not superior to ultrasonication or glass-bead milling, electroporation could be advantageous in the production of proteins where overexpressed native proteins challenge the host cell with toxicity or metabolic burden, as demonstrated in fed-batch production of α-amylase (Shiina et al., 2004). Extraction by means of electroporation was superior in terms of ratio between target protein and contaminating host protein. Furthermore, it was shown to largely avoid cell lysis and disintegration with which the extract is a mix of extracted proteins with debris of the bacterial envelope and bacterial DNA, which necessitates further purification. We expect that reduction of contaminants achieved by extraction by means of electroporation could lower the number of purification steps in the downstream process and decrease its costs.

We have also shown that the efficiency of extraction by means of electroporation is comparable in both electroporation systems (batch and flow through system), thus demonstrating feasible scale-up. Based on the successful application of electroporation in the food industry (Picart-Palmade et al., 2019), electroporation could be, owing to its flexibility and scalability, suitable for large scale protein production. The key benefit of electroporation is that is a non-heating technology with moderate energy consumption, thereby preventing unwanted effects of heat on final product. Furthermore, it can be implemented in any continuous production line (as sole technique or combined with other techniques) where thousands liters per hour are treated. Nevertheless, there are still some drawbacks and limitations, for instance high cost of the equipment, too high medium conductivity disables usage of electroporation, and optimization of the parameters are still needed (Martinez et al., 2020).

## Data Availability Statement

All datasets generated for this study are included in the article/Supplementary Material.

## Author Contributions

SHM and NJ drafted the manuscript, conceptualized the study, and conducted experiments. KF conducted experiments and developed the generator of electric pulses. DM and MP conceptualized the study and supervised the experimental work. DM and TK critically revised the manuscript, and improved the work with important intellectual content. All authors read and approved the submitted manuscript.

## Conflict of Interest

The authors declare that the research was conducted in the absence of any commercial or financial relationships that could be construed as a potential conflict of interest.

## Footnotes

1 https://www.edqm.eu/en/european-pharmacopoeia-ph-eur-9th-edition

## Supplementary Material

FIGURE S1 Flow treatment chamber used for continuous flow extraction by means of electroporation.
