# Supplementary figures and images for: Evaluation and Optimization of Protein Extraction From E. coli by Electroporation
Source: Front Bioeng Biotechnol. 2020 Sep 8;8:543187. doi: 10.3389/fbioe.2020.543187 (PMC7506034; doi:10.3389/fbioe.2020.543187)

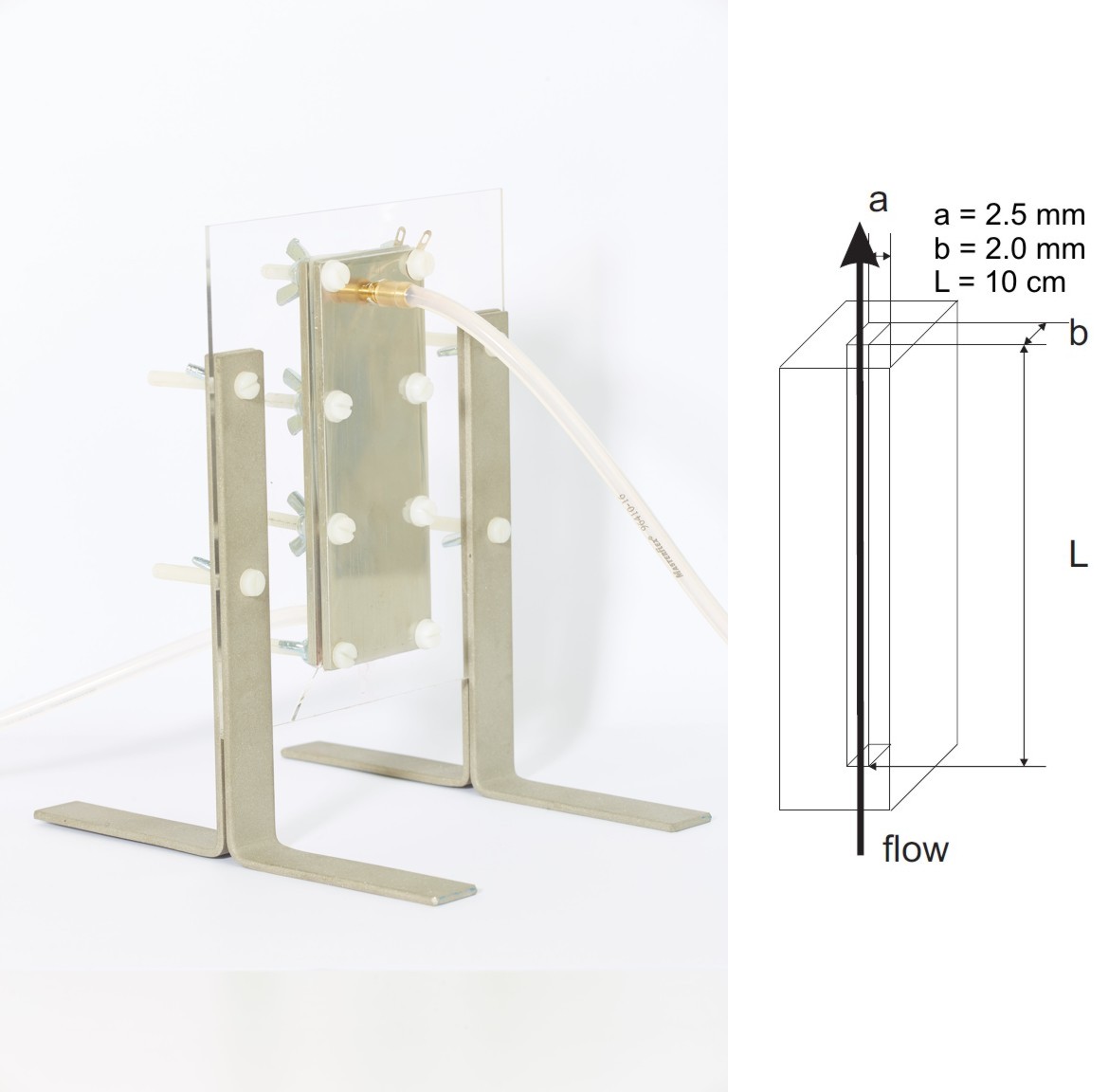

Supplement: FIGURE S1 — Flow treatment chamber used for continuous flow extraction by means of electroporation. [file Image_1.JPEG]
